# Editorials

**Published:** 1910-06

**Authors:** 


### EDITORIALS

The Business Office of the JOURNAL-RECORD is 1 1-2 to 5 1-2 South Broad Street.
The Editorial Office is 1014-15 Century Building.
Address all Business Communications to *Journal-Record of Medicine*, 1 1-2 to 5 1-2 South Broad Street.
Make remittances payable to *THE JOURNAL-RECORD OF MEDICINE.*
On matters pertaining to the Editorial and Original Communications, address Edgar G. Ballenger, M. D., Atlanta, Ga.
Reprints of Original Articles will be furnished at cost price. Requests for the same should always be made in THE MANUSCRIPT.
We will present, postpaid, on request, to each contributor of an original article, twenty (20) marked copies of THE JOURNAL-RECORD OF MEDICINE containing such article.

### PRESENT AND FORTHCOMING.

Those responsible for the mission of the JOURNAL-RECORD OF MEDICINE are naturally desirous that it should occupy a distinctive niche in Southern medical journalism.

Combined with a local flavor, we wish to also thoroughly cover a broad scientific field, appealing to every class of physicians, whether toiling in "the madding throng" of cities, or wrapped up in the strenuous life of the general practitioner in village or country.

Realizing the increasing demands for painstaking and accurate diagnosis, and all data pertaining thereto, we beg to announced that, beginning with this issue, we will be privileged to present to our readers a series of articles on present-day methods of diagnosis, both medical and surgical.

These studies will be produced by the facile pen of Dr. Lewis M. Gaines, of Atlanta, Ga., who we believe is fitted both by education and temperament to acceptably and entertainingly set forth the various diagnostic principles and methods so necessary to enlightened therapeutics.

We commend, therefore, to the thoughtful consideration of our readers, these careful efforts of Dr. Gaines.

